# Resolving Peto’s paradox: Modeling the potential effects of size‐related metabolic changes, and of the evolution of immune policing and cancer suppression

**DOI:** 10.1111/eva.12993

**Published:** 2020-06-04

**Authors:** Leonard Nunney

**Affiliations:** ^1^ Department of Evolution, Ecology, and Organismal Biology University of California Riverside Riverside CA USA

**Keywords:** blue whale, body size, cancer, cancer suppression, elephant, evolution, immune policing, multistage carcinogenesis, somatic mutation

## Abstract

The intrinsic risk of cancer increases with body size and longevity; however, big long‐lived species do not exhibit this increase, a contradiction named Peto's paradox. Five hypotheses potentially resolving this paradox were modeled using the multistage model of carcinogenesis. The five hypotheses were based on (1) intrinsic changes in metabolic rate with body size; adaptive increase in immune policing of (2) cancer cells or (3) cells with driver mutations; or adaptive increase in cancer suppression via (4) decreased somatic mutation rate, or (5) increased genetic control. Parameter changes needed to stabilize cancer risk in three types of cancer were estimated for tissues scaled from mouse size and longevity to human and blue whale levels. The metabolic rate hypothesis alone was rejected due to a conflict between the required interspecific effect with the observed intraspecific effect of size on cancer risk, but some metabolic change was optionally incorporated in the other models. Necessary parameter changes in immune policing and somatic mutation rate far exceeded values observed; however, natural selection increasing the genetic suppression of cancer was generally consistent with data. Such adaptive increases in genetic control of cancers in large and/or long‐lived animals raise the possibility that nonmodel animals will reveal novel anticancer mechanisms.

## 1 INTRODUCTION

The multistage model of carcinogenesis was proposed by Nordling (1953) and, despite a variety of suggested modifications (see Hornsby, Page, & Tomlinson, 2007), remains the model that underpins our understanding of how cancer initiates. The model assumes that a number of driver mutations (inherited and/or resulting from somatic mutation) must accumulate in a single cell for cancer to arise. A clear prediction of this model is that, if all else is equal, more somatic mutation will lead to an increased cancer risk, which, in turn, means that a large long‐lived organism such as a human should get more cancer than a small short‐lived one such as a mouse (Peto, 1977). However, the overall incidence of cancer in humans and mice is remarkably similar (Rangarajan & Weinberg, 2003), and more broadly, there is no evidence of an increase in cancer incidence as body size increases across a broad range of mammals (Abegglen et al., 2015). This contradiction between the model prediction and empirical data has become known as Peto's paradox (Nunney, 1999a).

The multistage model can be used to quantify the increase in risk resulting from a larger size and/or longer lifespan (Caulin, Graham, Wang, & Maley, 2015; Caulin & Maley, 2011; Nunney, 1999a, 2003). The basic model defines the accumulated risk of cancer (*p*) by age *t* as:

(1) $$p\;=\;1\;-\;{\left\{1-{\left[1\;-\;\exp\left(-ukt\right)\right]}^{M}\;\right\}}^{C}$$

which, under typical conditions of cancer risk with *p* small, can be accurately approximated by:

(2) $$p=C{\left(\mathrm{ukt}\right)}^{M}$$

given *C* at‐risk cells, dividing at a rate *k*/unit time, with cancer arising if one cell acquires *M* driver mutations given a somatic mutation rate *u* per division (Nunney, 1999a). More precisely, the formulae (1) and (2) assume that *u* is constant for all driver mutations and that all of the controlling genes are recessive, noting that *u* is not defined in terms of its precise nature of the mutation (e.g., base pair substitution, or epigenetic change) but more generally in terms of the probability of a driver mutation. Relaxing many of these simplifications can be incorporated easily into the equations and result only in minor changes (Nunney, 1999a). Thus, provided cancer is relatively rare, it is generally expected that the risk of cancer increases linearly with cell number (∝*C*) and even more strongly with longevity (∝*t^M^*).

Cairns (1975) recognized the danger of high levels of somatic mutation in rapidly renewing tissues (such as the epithelium) of long‐lived animals. He suggested this could be achieved if relatively few “immortal” stem cells were responsible for tissue renewal (minimizing *C*), that they retained the “old” DNA strand (minimizing *u*), and if stem cells were compartmentalized to minimize the potential for a mutant cell to outcompete its neighbors. These strategies are important, but are likely to be difficult to continuously scale as animals get ever larger and longer‐lived.

A similar recognition of the dangers of somatic mutation in large, long‐lived mammals led Burnet (1970) to develop the immunosurveillance hypothesis, suggesting an important role for the immune system in policing cancer cells. His view of the potential importance of the immune system was supported by the recognition of spontaneous regression of potentially lethal cancers, first emphasized more than 100 years ago by Coley (1893), and by the recognition of tumor‐specific antigens (Klein, 1966) that could be targeted. An implicit assumption of the immunosurveillance hypothesis is the possibility that increasing size or longevity could be associated with more effective policing of cancer cells through the evolution of the immune system.

It is clear that a wide range of mechanisms could in principle be modified to decrease in cancer risk (Caulin & Maley, 2011). The first general solution proposed to resolve Peto's paradox was via the adaptive evolution of cancer suppression through the recruitment of additional genes in the control of specific cancers. This adaptive strategy was formalized in an evolutionary model of multistage carcinogenesis (EMMC) (Nunney, 1999a, 2003). The model assumes that cancer suppression is an evolving trait and that suppression has the potential to increase whenever a fitness loss due to a specific cancer is large enough for natural selection to be effective. A “large enough” fitness loss is defined roughly by 1/(2*N_e_*), where *N_e_* is the effective population size (Wright, 1931). The specific nature of this adaptive response would depend upon the genetic variation present in the population. Thus, the response could involve the tissue‐specific recruitment of one or more additional tumor suppressor genes that directly reduces the incidence of the targeted cancer, or a more general response, such as the suppression of telomerase across the broad spectrum of tissues, a response that, in addition to reducing the incidence of the targeted cancer, could incidentally reduce the incidence of other types of cancer.

An alternative to the adaptive evolution of enhanced immune policing or cancer suppression as a species evolves to be larger and/or longer‐lived is the possibility that intrinsic life‐history scaling compensates for changes in cancer risk. In particular, it has been proposed that the decrease in cellular metabolic rate with body size can account for the resolution of Peto's paradox (Dang, 2015). Thus, there are three broad (but nonexclusive) categories that could be responsible for resolving Peto's paradox by keeping cancer risk relatively constant regardless of body size or longevity: nonadaptive scaling effects; adaptive cancer suppression; and adaptive immune policing (Table 1).

**TABLE 1:** Summary of the five hypotheses tested for their ability to resolve Peto's paradox

	Hypothesis	Parameter change	Reference equations
Intrinsic effect of size			
Metabolic rate change	MR: Lower somatic mutation rate and/or slower cell division	*uk* ↓	Equations 1, 4, 7, 8
Adaptation in response to size and/or longevity			
Suppression	Increased genetic suppression (more driver mutations)	*M* ↑	1, 2
	Decreased somatic mutation rate	*u* ↓	1, 2
Immune policing	Increased detection of cancer cells	*e_c_* ↑	5
	Increased detection of cells with driver mutations	*e_d_* ↑	6

The goal of this paper was first to examine the potential influence of the metabolic rate hypothesis in fully resolving Peto's paradox in light of the available data. If this possibility is strongly supported, then the adaptive explanations are likely to be moot. The second goal is to test the plausibility of four evolutionary hypotheses for controlling cancer risk, either with or without some level of metabolic rate effect. These evolutionary hypotheses involve adaptive changes either in cancer suppression via changes in (a) the somatic mutation rate, or (b) the number of driver mutations required to initiate a cancer, or in the policing of cancer cells via changes in (c) the immune surveillance of cancer cells, or (d) the immune surveillance of individual driver mutations. The multistage model (Equation 1) was used to quantify the potential effects of these various hypotheses on three different cancers during the theoretical transition from an organism with the size and longevity of a mouse, to one with the characteristics of a human, and of a blue whale.

## 2 MATERIALS AND METHODS

### 2.1 The metabolic rate hypothesis

The metabolic rate (MR) hypothesis is based on the long‐established relationship between total body basal metabolic rate and body weight. Across species there is a linear log‐log relationship between these variables with a slope of about 0.75 (Kleiber, 1947), although there has been a long‐standing debate over whether 3/4 or 2/3 is the most appropriate slope (Glazier, 2005). For example, both Speakman (2005) and de Magalhães, Costa, and Church (2007) obtained a slope of 0.71 based on 639 and 300 species of mammal, respectively. Thus, a good description of how whole‐body metabolic rate changes with size is provided by a log‐log slope of 0.7. The same general relationship also applies within mammal species, including humans and domestic dogs, and the exponent is generally within the range 0.5–0.75 (Glazier, 2005).

This whole‐body scaling exponent of 0.7 converts to a unit mass, and hence cellular, exponent of −0.3 by dividing through by body weight (*W*) (Savage et al., 2007) giving the change in cellular‐level basal metabolic rate (*R*) with body weight (*W*) as:

(3) $$R=W^{-0.3}$$

The MR hypothesis predicts that, due to the change in metabolic rate, the rate of accumulation of somatic mutations per cell lineage per unit time (=*uk*) declines with *C*, and as a result, the incidence of cancer does not increase with body size. Assuming that *uk* is proportional to *R*, and that tissue size (*C*) is proportional to body size (*W*) gives:

(4) $$uk=u^{\prime }k^{\prime }C^{-0.3}$$

where *u*’ and *k*’ are independent of size. This transform allows the MR hypothesis to be tested using the multistage model, by substituting Equation (4) into Equations (1) or (2).

To avoid confounding the MR hypothesis with the hypothesis that the somatic mutation rate *u* evolves in response to changing cancer risk, in the numerical examples, the effect of *R* on *uk* is mediated through changes in *k*. This has no effect on the results since *u* and *k* only occur as their product in Equations (1) and (2).

### 2.2 Testing hypotheses

The magnitude of the change in cancer risk under the MR hypothesis and the four different evolutionary hypotheses was tested using differences in size and longevity among three different species, the house mouse (*Mus musculus*), humans (*Homo sapiens*), and the blue whale (*Balaenoptera musculus*), by assigning respective mean weights of 0.02, 60, and 150,000 kg and lifespans of 2, 80, and 90 years. It was assumed that the cell number in different tissues scale in proportion to body mass.

Three types of cancer were chosen to illustrate these scaling effects, colorectal adenocarcinoma, esophageal squamous cell carcinoma, and hepatocellular carcinoma. These cancers represent a broad range of parameter values: Two are derived from large stem cell populations that differ in having a high (colorectal) or low (hepatocellular) rate of stem cell division, while the third (esophageal) is derived from a much smaller stem cell population with an intermediate rate of cell division. For each of these cancers, there are estimates from humans on the size and rate of division of the underlying stem cell populations: colorectal with 2 × 10^8^ stem cells dividing 73 times per year; hepatocellular with 3.01 × 10^8^ stem cells dividing 0.9125 times per year; and esophageal with 6.6528 × 10^6^ stem cells dividing 33.2 times per year (Tomasetti, Li, & Vogelstein, 2017; Tomasetti & Vogelstein, 2015).

The mouse was used to provide a baseline for comparisons across the three species. First, the appropriate number of stem cells in the mouse was estimated from the human data assuming a proportional change in number with body size. Second, a rate of stem cell division for each of the three tissues was assigned to the hypothetical mouse based on the human values modified according to three hypotheses of size‐related change: no change, change in *uk* proportional to the change in metabolic rate (i.e., using a body size scaling exponent of −0.3), and an intermediate rate using a body size scaling exponent of −0.15. Third, a hypothetical lifetime frequency of 1% for each cancer was used to estimate the two remaining parameters for each cancer using Equation (1): An integer number of driver mutations (*M* ≥ 2) was combined with a somatic mutation rate to bring the lifetime risk in the hypothetical mouse to 1%. In order to keep the somatic rates similar across tissues, *M* was chosen to keep the rate *u* as close as possible to 10^−5^/cell division.

To evaluate the MR hypothesis, each of the nine baseline models (three cancer types with the three different scaling exponents: 0, −0.15, and −0.3) was used to estimate the risk of cancer in the two larger, longer‐lived species assuming no evolved parameter changes.

To evaluate the four evolutionary hypotheses, in each case it was determined what parameter shifts were needed to bring the lifetime cancer risk in the two larger mammals back down to 1%. For the somatic mutation and the driver mutation hypotheses, this involved, respectively, determining the required fold reduction in *u*, and the required increase in *M*. For the two immunosurveillance hypotheses, it required determining the value of a new parameter (=*e*) measuring the effectiveness of the immune system at detecting and eliminating either (i) cancer cells (cells with all *M* required driver mutations) or (ii) cells that had acquired any driver mutation. These were modeled by adding the parameter *e* to Equation (1) either as *e_c_*:

(5) $$p=\;1-{\left\{1-\left(1-e_{c}\right){\left[1-\exp\left(-ukt\right)\right]}^{M}\right\}}^{C}$$

for the case of the immunosuppression of cancer cells, and *e_d_*:

(6) $$p=\;1-{\left\{1-{\left[\left(1-e_{d}\right)\left(1-\exp\left(-ukt\right)\right)\right]}^{M}\right\}}^{C}$$

for the case of the immunosuppression of any cell with a driver mutation.

## 3 RESULTS

The hypotheses tested are summarized in Table 1.

### 3.1 Metabolic rate scaling

The MR hypothesis invokes an intrinsic effect of size and therefore applies both within and between species. Within a species, the effect of size differences on the lifetime incidence of cancer under the MR hypothesis (defined by Equation 4) can be written as:

(7) $$p\;=\;C{\left(u^{\prime }k^{\prime }C^{-0.3}T\right)}^{M}\;=\;C^{\left(1-0.3M\right)}\;{\left(u^{\prime }k^{\prime }T\right)}^{M}$$

given a constant lifespan *T*, noting that the relatively small intraspecific changes in size allow the use of Equation (2). Equation (7) shows that the effect of size (*C*) on cancer risk is dependent upon the number of driver mutations *M*; however, it is well established that *M* varies across cancer types in humans, ranging from a low of 2 in retinoblastoma (Knudson, 1971) to perhaps as high as 10 or more in other cancers (Martincorena et al., 2017). Based on Equation (7), it follows that as body size (reflected in *C*) increases, the incidence of cancers with *M* = 2 increases, while incidence is largely unaffected for those with *M* = 3, and reduced for those with *M* ≥ 4. Therefore, given that typically *M* > 2, the MR hypothesis is expected to result in larger individuals within a species having about the same or lower incidence of cancer than small individuals. However, this pattern is not supported by the data. Cancer risk increases with body size across breeds of domestic dog (Fleming, Creevy, & Promislow, 2011; Nunney, 2013) and, in humans, an increase in proportion with body size is observed across a broad range of cancers (Nunney, 2018). Thus, the hypothesis that somatic mutations (and hence driver mutations) accumulate at a rate proportional to metabolic rate is only supported if, in general, *M* = 2; however, in humans the average value of *M* has been estimated conservatively to be about 4 (Martincorena et al., 2017).

In Equation (7), it was assumed that *T* is independent of size. This independence is broadly true in humans, although there is evidence that taller humans have a shorter lifespan (Samarasa, Elrick, & Storms, 2003), while this is well established in domestic dogs: In general, the lifespan of large dog breeds is substantially shorter than that of small breeds (Kraus, Pavard, & Promislow, 2013; Michell, 1999). Incorporating a shorter lifespan of larger individuals into Equation (7) would strengthen the MR hypothesis prediction that larger individuals would have a lower lifetime cancer risk, a prediction known to be wrong.

To evaluate the interspecific pattern predicted by the same MR scaling (=*C* ^−0.3^) requires the inclusion of the effect of size on lifespan *T*. Size and lifespan are typically positively correlated across species, with a linear log‐log relationship having a slope of about 0.3 (Speakman, 2005). This correlation suggests that metabolic rate could scale with lifespan as well as size; however, after correcting for the effect of body size, this is not the case (de Magalhães et al., 2007).

The lifespan/body size relationship (*T* = *T*'*C* ^0.3^), incorporated into Equation (4), shows that the product u*kT* is size independent (=*u*'*k*'*T*'). Thus, the MR hypothesis predicts that as size increases, cancer risk (*p*) increases in proportion to *C* when *p* is small (Equation 2):

(8) $$p\;=\;C{\left(u^{\prime }k^{\prime }T^{\prime }\right)}^{M}$$

since *u*’, *k’, and T*’ are constants independent of body size, with *p* asymptotically approaching unity (as defined by Equation 1) as *C* continues to increase.

This dependence upon *C* predicts that large long‐lived mammals would have a much higher lifetime risk of cancer than smaller short‐lived ones. This effect can be quantified using the model‐based transition from a mouse to a human or blue whale (Table 2c), which shows that the lifetime incidence of all three of the cancer types (colorectal, hepatocellular, and esophageal) increases from the 1% that was set as a baseline in the mouse to 100% in the two larger species. Consequently, a weaker MR effect (with a scaling exponent of −0.15) yields the same result (Table 2b), as does the absence of any MR effect (Table 2a).

**TABLE 2:** The scaling of cancer risk from mouse to human and to blue whale assuming different metabolic rate (MR) size corrections: (a) no size effect on the division rate, (b) a size‐related reduction in the division rate (*k*) defined by *C* ^−0.15^, and (c) a size‐related reduction of *k* ∝ *C* ^−0.3^

			No change versus mouse				Somatic mutation rate		Immune policing of:				Suppression by drivers
									Cancers		Driver mutations		
			Lifetime risk				Parameter changes needed for a lifetime risk = 1% Baseline u/"?" probability of target cell escaping added M						
(a) No MR correction; *k* constant.													
Colorectal adenocarcinoma (large stem cell population, high division rate)													
Mouse baseline: *M* = 3; *u* = 3.66E−05													
		Human	100%			578			7.10E−09		1.93E−03		11.4
		Whale	100%			8,820			2.10E−12		1.28E−04		17.4
Hepatocellular carcinoma (large stem cell population, low division rate)													
Mouse baseline: *M* = 2; *u* = 1.74E−04													
		Human	100%		2,180				2.10E−07		4.60E−04		3.5
		Whale	100%		123,000				6.80E−11		8.20E−06		5.5
Esophageal squamous cell carcinoma (small stem cell population, intermediate division rate)													
Mouse baseline: *M* = 2; *u* = 3.24E−05													
		Human	100%	2,200				2.30E−07		4.80E−04		6.1	
		Whale	100%	124,000				7.40E−11		8.50E−06		9.8	
(b) MR correction; *k* varies by *C* ^−0.15^.													
Colorectal adenocarcinoma (large stem cell population, high division rate)													
Mouse baseline: *M* = 3; *u* = 1.10E−05													
		Human	100%	174				2.10E−07		5.95E−03		5.5	
		Whale	100%	920				1.90E−09		1.24E−03		5.3	
Hepatocellular carcinoma (large stem cell population, low division rate)													
Mouse baseline: *M* = 2; *u* = 5.27E−05													
		Human	100%	660				2.30E−06		1.51E−03		2.3	
		Whale	100%	3,450				7.60E−09		8.70E−05		2.8	
Esophageal squamous cell carcinoma (small stem cell population, intermediate division rate)													
Mouse baseline: *M* = 2; *u* = 1.32E−05													
		Human	100%	900				1.30E−06		1.14E−03		4.0	
		Whale	100%	27,100				4.30E−09		6.50E−05		4.4	
(C) MR correction; *k* varies by *C* ^−0.3^.													
Colorectal adenocarcinoma (large stem cell population, high division rate)													
Mouse baseline: *M* = 4; u = 1.24E−05													
		Human	100%	27				2.2E−06		3.8E−02		4.9	
		Whale	100%	20				5.6E−06		4.9E−02		2.5	
Hepatocellular carcinoma (large stem cell population, low division rate)													
Mouse baseline: *M* = 2; u = 1.58E−05													
		Human	100%	198				2.6E−05		5.1E−03		1.6	
		Whale	100%	1,070				8.8E−07		9.4E−04		1.6	
Esophageal squamous cell carcinoma (small stem cell population, intermediate division rate)													
Mouse baseline: *M* = 3; u = 2.28E−05													
		Human	100%	52				7.6E−06		2.0E−02		4.2	
		Whale	100%	77				2.3E−06		1.3E−02		2.6	

The baseline parameters for the mouse (*M* and *u*) were calculated as described in the text. The required change in the somatic mutation rate (*u*) shown is the divisor of the mouse value, for the immune policing it is the probability of a targeted cell escaping (assuming a value of 1.0 for the mouse), and for suppression by drivers, it is the number of extra driver mutations added (relative to the mouse)

In summary, current evidence suggests that the MR hypothesis alone cannot resolve Peto's paradox. Modeling showed that the interspecific effect of an MR‐mediated reduction in cancer risk scaling as *C* ^−0.3^ (or less) will, on average, result in an initial linear (or greater) increase in cancer risk with body size when risk is low and it is therefore insufficient to resolve Peto's paradox. The intraspecific data are inconsistent with a scaling of *C* ^−0.3^ or more. In fact, the intraspecific data are most consistent there being no effect of size‐related changes in metabolic rate on cancer risk, although the possibility of a small effect cannot be excluded. For this reason, in evaluating the evolutionary hypotheses, two possible baselines were considered to be viable options: (i) no effect and (ii) a body size scaling of *C* ^−0.15^, which defines a rough upper limit to a scaling that could be consistent with the intraspecific data. The results for a scaling of *C* ^−0.3^ are also included for reference (Table 2c).

### 3.2 Cancer suppression as an evolving trait

The evolutionary model of multistage carcinogenesis (EMMC) was developed based on the assumption that the recruitment of additional layers of suppression was the primary way in which adaptive evolution resolved Peto's paradox (Nunney, 1999a, 2003). In the model, this adaptation involved the recruitment of extra recessive tumor suppressor genes (adding two extra driver mutations) or dominant protooncogenes (adding one driver mutation) able to further limit the occurrence of a cancer whenever that cancer was causing a significant average loss of fitness in a species. This recruitment of extra layers of control could involve the duplication of one or more preexisting controlling genes or through the tissue‐specific recruitment (via increased expression) of preexisting but previously uninvolved genes. The question then becomes how many added driver mutations would be necessary for a given increase in size and/or longevity.

**FIGURE 1:**
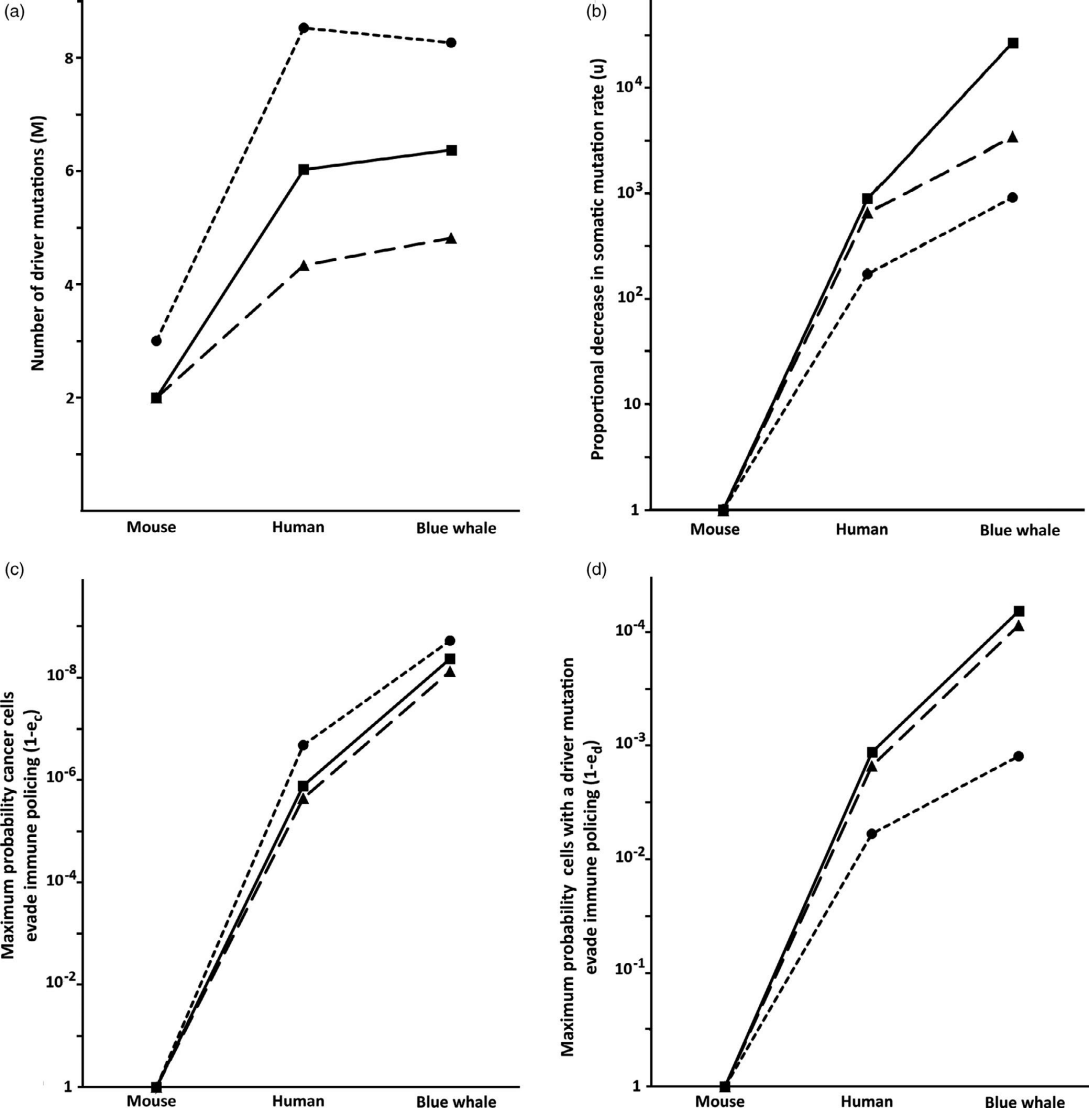
The adaptation required to stabilize cancer risk for three types of cancer given a transition from the size and longevity of a mouse to those of a human and a blue whale. Each of the four evolutionary hypotheses is shown, assuming an intermediate level of metabolic rate scaling (=*C* ^−0.15^): (a) genetic suppression, showing the total number of driver mutations required (i.e., the mouse baseline plus the required additions; see Table 2); (b) somatic mutation rate, showing on a log scale the magnitude of the decrease required; (c) immune detection of cancer cells, showing on a log scale the maximum level of cellular escape ((1‐*e_c_*); Equation 5) consistent with control; (d) immune detection of cells with driver mutations, showing on a log scale the maximum level of cellular escape ((1‐*e_d_*); Equation 6) consistent with control. The three types of cancer are colorectal (dotted line), with many stem cells and a high division rate; esophageal (solid line), with few stem cells and intermediate division rate; and hepatocellular (dashed line), with many stem cells and a slow division rate

In the absence of any MR effect (Table 2a), the transition to human size and longevity requires added suppression needing approximately 4 (hepatocellular carcinoma) to 12 (colorectal adenocarcinoma) disabling mutations. For the blue whale, this number increased to approximately 6 (hepatocellular carcinoma) to 18 (colorectal adenocarcinoma). The intermediate MR effect (Table 2b) has the effect of both reducing the number of added driver mutations required and, because the longevity of the blue whale is similar to that of humans (noting that the MR effect tends to correct for body size differences but not longevity differences), of equalizing the number required for humans and blue whales, ranging from approximately 3 (hepatocellular carcinoma) to 6 (colorectal adenocarcinoma) (Figure 1a).

It can be seen that the number of extra genetic controls required for each type of cancer is driven primarily by the rapidity of stem cell divisions (with colorectal > esophageal > hepatocellular; Table 1; Figure 1a).

### 3.3 Somatic mutation rate

A potentially powerful mechanism for resolving Peto's paradox is through an adaptive decrease in the somatic mutation rate with increasing body size and/or greater longevity. Under this hypothesis, *u* is an evolving species‐specific trait.

To bring the human cancer rates down to the 1% level would require a more than 2,000‐fold reduction to control all three cancers in the absence of an MR effect (Table 2a). The required reduction is substantially less for the control of colorectal adenocarcinoma, because the effect of changes in the somatic mutation rate has its effect as *u^M^*, and *M* is greater for colorectal adenocarcinoma. As might be expected, the reduction in the blue whale would need to be substantially greater. In the absence of a MR effect, a more than 120,000‐fold reduction is required to control all three cancers (Table 2a).

Given an MR size effect of *C* ^−0.15^, the somatic mutation rate would still need to be reduced in humans by 900‐fold to control all three cancers and by more than 27,000 in the blue whale (Table 2b). Under this model, each of the three cancers responds differently, with esophageal squamous cell carcinoma requiring a greater evolutionary response than the other two cancers to achieve successful control, a difference that is amplified in the blue whale (Figure 1b).

### 3.4 Immune system cancer surveillance

To evaluate the potential role of the immune system in mitigating the effect of increased size and longevity, its effect was first modeled assuming that the immune system, with some efficiency *e_c_*, detects and destroys cancer cells that have accumulated all *M* of the driver mutations required to initiate malignancy. This effect is defined by Equation (5), which simplifies to *p* = *C*(1*‐e_c_*)(*ukt*)*^M^* for small *p*.

In the absence of any MR effect (Table 2a), in humans and in whales the system would have to detect essentially all cells that become cancerous. Assuming that the mouse immune system cannot detect any cancer cells, the human immune system would need to detect all but 7 in a billion cancer cells to control colorectal adenocarcinoma at the 1% lifetime level and all but about 2 in 10^7^ cancer cells for the other two cancers. These numbers become even more extreme in the case of the blue whale, at levels of all but 2 in a trillion for colorectal adenocarcinoma and all but about seven in one hundred billion for the other two cancers. The situation is less extreme given the intermediate MR effect (=*C* ^−0.15^; Table 2b), but it still requires the human immune system to detect all but 2 in ten million, and the whale to detect all but 2 in a billion cancer cells to control all three cancers. Under this model, the required response is similar across all three cancers (Figure 1c).

Perhaps the ideal scenario for immune system control of cancer is the possibility that it is capable of detecting and destroying (with some efficiency *e_d_*) cells carrying at least one driver mutation. This possibility is defined by Equation (6), or *p* = *C*[(1*‐e_d_*)(*ukt*)]*^M^* for small *p*. It is certainly possible that the immune system could detect some of these mutational changes (although probably not all). For example, p53‐specific antibodies have been detected in response to the elevated expression of p53 associated with many tumors (Soussi, 2000). However, even given the ability to detect a cell with a single driver mutation, Equation (6) shows that detection efficiency would have to be very high. Assuming no MR effect (Table 2a), and again assuming no detection ability in the mouse, humans would have to detect all but 5 in 10,000 driver mutations to control all three cancers, which corresponds to a 99.95% efficiency at destroying such cells, and for the blue whale the equivalent efficiency is 99.9992%.

Given the intermediate MR effect (Table 2b), the efficiency required by the human immune system to detect cells with driver mutations would still need to be around 99.9% to control all three cancers, while for the blue whale would need an efficiency of 99.993%. However, not surprisingly, the efficiency can be notably less when the number of driver mutations is greater, an effect seen for colorectal cancer versus the other two types examined (Figure 1d).

## 4 DISCUSSION

### 4.1 Peto's paradox

The predicted increase in cancer risk with size and longevity was recognized by Peto (1977) in his review of the multistage model, and, given that it was clear this did not happen (as quantified much more recently by Abegglen et al., 2015), he concluded that “Presumably some concomitant of our evolved ability to grow big and live for threescore years and ten is involved.” (Peto, 1977, p1414). This expected increase was illustrated by the null model lacking any metabolic effect or adaptive change as body size and longevity increased (Table 2a), where a 1% lifetime cancer risk in a mouse translated to a 100% risk when the longevity and size of a human or blue whale were imposed (see also Caulin et al., 2015; Caulin & Maley, 2011; Nunney, 1999a, 2003).

Nunney (1999a) suggested that the solution to Peto's paradox was adaptive evolution; however, this prediction would be incorrect if some intrinsic feature of being big and long‐lived, such as the metabolic rate effect, fortuitously corrected the problem.

### 4.2 The metabolic rate hypothesis

Dang (2015) proposed that the well‐established relationship between metabolic rate and body size, sometimes referred to a Kleiber's law (Kleiber, 1947), could resolve Peto's paradox. A test of the metabolic rate (MR) hypothesis showed that this is not the case. The MR hypothesis was tested using a size‐related exponent of −0.3 to define the drop in the accumulation of somatic mutations per unit time (=*uk*; see Equation 4), based on the assumption that the change in the rate at which somatic mutations accumulate over time is proportional to the change in cellular metabolic rate with body size (Equation 3). However, this value fails to stabilize cancer risk against interspecific changes in size, with an initially low cancer risk increasing linearly with body size (Equation 8). This failure is due to the effect of longevity increasing with body size, and the effect can be seen in the three cancers modeled, where lifetime risk still increased from 1% in the mouse to 100% given human or blue whale size and longevity (Table 2c).

It could be argued that perhaps the negative link between the somatic mutation rate and body size is even stronger than the link between cellular metabolic rate and body size; however, such a strong relationship is incompatible with the intraspecific data. It would result in an intraspecific decline in cancer risk with size, conflicting with the well‐established observation of the exact opposite in humans and domestic dogs, where an increase in risk with body size is seen (Fleming et al., 2011; Nunney, 2013, 2018). This intraspecific pattern is important because it excludes the influence of adaptive evolutionary change in driving the size‐related effect (Nunney, 2018).

Even though the available data indicate that the MR hypothesis cannot resolve Peto's paradox, it could still make a partial contribution. Therefore, it is still worth asking whether the decreased cellular metabolic rate associated with increased size does in fact lead to some overall decrease in the rate at which mutations accumulate. Savage et al. (2007) suggested that the energy change associated with size‐related shifts in metabolic rate are consistent with a change in the rate of cell division (*k*), and there is some evidence that the products of DNA damage produced per unit time decrease with size, based on a limited phylogenetic comparison of rodents and primates (Adelman, Saul, & Ames, 1988; Shigenaga, Gimeno, & Ames, 1989). However, perhaps the best evidence is from Milholland et al. (2017), who used sequence data to directly estimate the somatic mutation rate (*u*), finding that in mice it was 3x greater than that in humans. Given their size difference, this change is equivalent to a scaling exponent of −0.14. Notwithstanding that this difference could be due to adaptive evolution rather than intrinsic size‐related scaling, the evolutionary hypotheses were tested with a similar “intermediate” MR/body size scaling exponent of −0.15, as well as being tested assuming no MR effect at all.

### 4.3 Adaptive evolution

Cancer develops as a result of natural selection favoring the unregulated division of cells within an individual, selection that has the incidental effect of lowering the fitness of the multicellular individual within whom the cancer occurs. Moreover, if all else is equal, selection acting at the shortest timescale is the most effective, creating an evolutionary problem: How can the short‐term advantage of cancer cells be overcome by selection acting on a long‐lived individual. Fortunately, this conflict can be resolved through lineage selection (Nunney, 1999a, 1999b, 2017). While the intrinsic advantage of cancer cells cannot be changed, lineage selection can act at the individual level to enhance cancer suppression and policing.

Four adaptive evolutionary hypotheses were tested for their potential role in stabilizing cancer risk against increasing body size and longevity, two involving increased policing by the immune system in its ability (i) to detect cancer cells or (ii) to detect cells with at least one driver mutation, and two involving increased suppression, either (iii) through decreasing the somatic mutation rate or (iv) through the recruitment of added levels of genetic control inhibiting the initiation of cancer. Of these, increased genetic control was the hypothesis that, based on current data, appears to be the most plausible. The degree of increased immune efficiency required to stabilize cancer risk was found to be substantial, going far beyond levels indicated by current data (see below). The reduction in somatic mutation rate needed was also far in excess of levels documented.

The potential role of the immune system in resolving Peto's paradox depends upon the answer to two questions. First, how important is immune system policing in detecting and eliminating cancers as they arise? Second, does the effectiveness of this policing role increase with body size? The first question has been the subject of much debate ever since the beginning of the last century after Coley (1893) found that streptococcal infection following surgery could sometimes stimulate the immune system and result in tumor regression. However, his ideas did not gain general acceptance and it was not until half a century later that they resurfaced when Burnet (1957) suggested that the unique antigenic properties of cancer cells might provoke an immunological reaction and Thomas (1959) proposed that the immunological response to such tumor antigens represented an important natural defense against cancer. These ideas led to the formulation of the cancer immunosurveillance hypothesis (Burnet, 1970), based on his recognition that in large long‐lived animals, somatic mutations necessary for the initiation of cancer would be relatively common. Burnet (1970, p3) argued that “It is an evolutionary necessity that there should be some mechanism for eliminating or inactivating such potentially dangerous mutant cells and it is postulated that this mechanism is of immunological character.” One of the strongest pieces of evidence in support of the immunosurveillance hypothesis related back to Coley’s (1893) work: the rare but well‐established observation of the spontaneous regression of malignant tumors (Everson, 1964).

The immunosurveillance hypothesis was generally ignored after the 1970s, until interest was revived in the early 2000s when evidence began to accumulate demonstrating that immunodeficient mice were more prone to developing tumors than their immunocompetent controls (see Dunn, Koebel, & Schreiber, 2006). This included the finding that tumors from immunodeficient mice were rejected when transplanted into immunocompetent mice, but tumors from other immunocompetent mice were not, suggesting that the immune system was acting as a selective agent to limit or alter the immunogenicity of tumor cells. These observations led to a modified form of the immunosurveillance hypothesis termed immunoediting, consisting of three phases: elimination; equilibrium; and escape (Dunn, Bruce, Ikeda, Old, & Schreiber, 2002). However, this hypothesis leaves open the question of what proportion of potential malignancies the immune system eliminates. This question can be examined using data on immunocompromised humans. If the immune system is important in reducing the incidence of cancer, then a clear prediction is that immunosuppressed individuals should be at a significantly higher risk of cancer.

Immunosuppression does result in an increased incidence of cancer, as seen in patients with AIDS (Patel et al., 2008) and in patients who are immunosuppressed following organ transplantation (Engels et al., 2011); however, the increase is primarily in cancers known to be promoted by viral infection. For example, increases in Kaposi sarcoma of > 50‐fold and in non‐Hodgkin lymphoma of > 7‐fold are seen in both groups. In contrast, the increase in cancers with no known link to infection is typically modest. In organ transplant recipients in the United States, the increase in noninfection related malignancies was found to be only 1.7‐fold (Engels et al., 2011). This result is consistent with the immunoediting hypothesis, in which cancers ultimately escape from the effect of immune policing. Thus, it appears that the effectiveness of the immune system is limited by the strong selection acting on the population of cells within a malignancy favoring those that are capable of avoiding or of local inhibition of the immune attack. Unfortunately, there are many mechanisms by which this can be achieved (Mellman, Coukos, & Dranoff, 2011).

It is clear from these human data that risk reduction by the immune system of cancers not linked to pathogen infection, while notable, is very limited. This result argues against immune policing providing a solution to Peto's paradox. Furthermore, there is only very limited counter evidence. For example, while there is evidence that the costs associated with the immune system across species decrease as body size increases, contrasting with this is the finding that the costs increase with longevity; in any event, the magnitude of these effects is small (Brace et al., 2017). Similarly, it is clear that there are differences among the immune system of mammalian species (Haley, 2003), with differences between the two best studies species, mice and humans, well documented (Mestas & Hughes, 2004); however, the extent to which any of these differences relate to the detection of cancer cells is unknown.

The models indicated that compared to a mouse, the ability of the human immune system to eliminate would have to be extraordinarily efficient. Even given the assistance of an intermediate metabolic rate effect (Table 2b), if the immune system of the mouse was 0% efficient at detecting cancer cells, the human immune system would have to be 99.99998% efficient, and the blue whale even more so, to limit all three cancers modeled to a 1% lifetime risk. Alternatively, invoking the most effective system of policing, whereby the immune system evolves to detect any cell with at least one driver mutation, then, again with the assistance of the intermediate metabolic rate effect, the immune system would have to evolve from a 0% efficiency in the mouse to a 99.9% efficiency in humans and a 99.993% in the blue whale (Table 2b). Unfortunately, there is no evidence that supports the possibility that the immune systems of large mammals can detect cancer cells with an enhanced level of effectiveness. Instead, the data on immunosuppressed individuals suggest that the immune system generally has a relatively minor role in controlling the onset of cancer (Engels et al., 2011; Patel et al., 2008).

But what might limit the effectiveness of natural selection in increasing the power of immunosurveillance? One possibility is the classic trade‐off between effectiveness and energetic cost; however, a much more relevant and important problem is autoimmunity. Tumor‐specific antigens are typically closely related to self‐antigens, making it very difficult for the immune system to recognize one but not the other. For example, paraneoplastic autoimmune disorders (PND) arise from cross‐reactivity between an antitumor immune response and neurologic antigens (Albert & Darnell, 2004), and immunotherapy using immune checkpoint inhibitors frequently results in immune‐related adverse events (irAEs; see Kumar et al., 2017; Myers, 2018). These effects suggest that increasing the efficiency of immune detection by the large amounts required to compensate for increases in size and longevity is unlikely, consistent with the data from immunosuppressed individuals.

The necessity for large degrees of change was also found when the somatic mutation rate was assumed to be the factor keeping cancer risk stable across changes in size and/or longevity. While the comparison of humans relative to mice revealed a threefold drop in the somatic mutation rate (Milholland et al., 2017), the model with intermediate MR required a 900‐fold reduction, and with no MR effect, the drop needed would be >2,000‐fold (Table 2a,b).

In contrast to the large adaptive responses needed in improving the immune system or in limiting somatic mutation, the models showed that the number of extra genetic controls needed was more realistic. The results illustrated two important predictions of the EMMC. First, that the predicted level of control varies across different tissues, with large rapidly dividing tissues (as in colorectal carcinoma) requiring more additional regulation, and second that, in terms of recessive controllers such as tumor suppressor genes, the number of genes (each representing 2 driver mutations) that needed to be recruited is relatively small, ranging from 2 (hepatocellular carcinoma) to 6 (colorectal adenocarcinoma) assuming no effect of metabolic rate on the accumulation of mutations (Table 2a), with these numbers reduced to just 1.5 to 3 if metabolic rate/size changes modestly reduce the accumulation of somatic mutations (Table 2b). In line with these numbers, Rangarajan, Hong, Gifford, and Weinberg (2004) showed that, while just two pathways needed to be perturbed to immortalize mouse fibroblasts, human fibroblasts required an additional four pathways to be modified.

There is a growing body of data that is consistent with the evolution of cancer suppression via the recruitment of added layers of genetic control. The most compelling is the finding that the degree of telomerase suppression in fibroblasts of 15 rodent species is strongly correlated with body size, with the largest (and unrelated) species in the study, the capybara and the beaver, showing the greatest level of suppression (Seluanov et al., 2007). In single species, other mechanisms identified include enhanced contact inhibition seen in fibroblasts of the long‐lived naked mole rats (Seluanov et al., 2009), and a massive necrotic response in fibroblasts to overproliferation in similarly long‐lived (but unrelated) blind mole rats (Gorbunova et al., 2012). These examples suggest that the enhanced control of specific cancers can be achieved by the recruitment of existing genes not previously involved in suppression in the target tissue (Nunney, 2003). Tumor suppressor genes and protooncogenes are not equally expressed across tissues and it has been shown that in humans high expression levels of these genes are associated with active involvement in cancer suppression (Muir & Nunney, 2015), supporting the hypothesis that tissue‐specific increases in the expression levels of cancer‐related genes can enhance suppression.

Gene duplication is another potential mechanism by which cancer suppression could be enhanced (Caulin & Maley, 2011; Nunney, 2003), and Caulin et al. (2015) adopted a general approach of searching the available genomes for duplicated cancer‐related genes. Analysis of the elephant genome, combined with cell‐culture experimentation, revealed an enhanced sensitivity of elephant cells to double‐stranded DNA breaks (leading to apoptosis) that appears to be linked to the finding of multiple retrogene copies of Tp53 (Abegglen et al., 2015; Sulak et al., 2016) and to the duplicated LIF gene (Vazquez, Sulak, Chigurupati, & Lynch, 2018). There is also evidence of the duplication of cancer‐related genes linked to apoptosis in whales, the only mammals that are even larger than the elephant (Tollis et al., 2019).

Another possibility is that cancer suppression could be enhanced by altering a preexisting gene via amino acid changes, which would leave a signal of positive selection. The extent to which such enhancement could mitigate against the effect of somatic mutations is unclear; however, evidence of positive selection in cancer‐related genes has been reported in a number of studies (Morgan et al., 2012; O’Connell, 2010; Tollis et al., 2019; Vicens & Posada, 2018).

Each of the adaptive hypotheses was considered alone and in combination with some degree of metabolic rate effect. At present, there is no direct evidence supporting a metabolic rate effect; however, it is certainly a possibility, and including a maximum level of effect that is reasonably consistent with the data (*C* ^−0.15^) shows that it can markedly reduce the adaptive response needed (Table 2a versus Table 2b). It is also possible that more than one of the evolutionary hypotheses could be combined in the overall adaptive response to size and longevity changes. Three factors will influence the relative importance of each of the types of response. First is the fitness cost of any response. Cost might be a particular problem for immune policing, if it induces some risk of autoimmunity. Similarly, there is good evidence that it is costly to reduce the germline mutation rate (Maklakov & Immler, 2016), and it is reasonable to expect that similar costs apply to the somatic mutation rate. Second is the availability of the appropriate genetic variation, and third is the fitness gain that favors a novel beneficial allele. A priori there is no reason why a lack of variation might adversely affect the different hypotheses; however, the effect size of beneficial mutations might be expected to differ, and beneficial alleles with a very small fitness effect are unlikely to spread. Variation decreasing the somatic mutation rate or increasing immune policing might be expected to be multigenic, with single alleles of large effect unusual. Of course, this is speculative; however, less speculative is the likelihood that a single genetic change will be able to change the tissue‐specific expression of a preexisting tumor suppressor gene so that it adds to the level of tumor suppression in the at‐risk tissue (while still retaining its similar preexisting role in other tissues), and modeling shows that such changes can effectively spread in a population in response to a change in body size (Nunney, 2003). However, the ultimate need is for more comparative data that can shed light on the reality or otherwise of these arguments.

In conclusion, empirical evidence combined with modeling using the multistage model supports the view that, while size‐related changes in metabolic rate may affect cancer risk, these changes do not resolve Peto's paradox. Thus, adaptive changes are needed, and of the hypotheses tested, the one that seems likely to dominate in this adaptive response is the one built into the evolutionary model of multistage carcinogenesis (EMMC): that the incidence of cancer is regulated primarily through the recruitment of additional layers of genetic control whenever a cancer significantly reduces average fitness (Nunney, 1999a, 2003). The specific controls recruited would depend upon the genetic variation present at the time, so that the same cancer could be regulated by somewhat different mechanisms in different species, and so could different cancers within the same species. It is to be hoped that recognizing the importance of evolution in modifying cancer risk in different ways will promote the study of nonmodel animals, with the goal of revealing potentially useful mechanisms of cancer suppression (Nunney, Maley, Breen, Hochberg, & Schiffman, 2015; Seluanov, Gladyshev, Vijg, & Gorbunova, 2018; Tollis, Schiffman, & Boddy, 2017).

## CONFLICT OF INTEREST

None declared.

## Data Availability

All data and formulae used are included in the body of the manuscript.
